# How emotions affect logical reasoning: evidence from experiments with mood-manipulated participants, spider phobics, and people with exam anxiety

**DOI:** 10.3389/fpsyg.2014.00570

**Published:** 2014-06-10

**Authors:** Nadine Jung, Christina Wranke, Kai Hamburger, Markus Knauff

**Affiliations:** Experimental Psychology and Cognitive Science, Justus Liebig UniversityGiessen, Germany

**Keywords:** logical reasoning, emotions, conditional reasoning, Wason selection task, spider phobia, exam anxiety

## Abstract

Recent experimental studies show that emotions can have a significant effect on the way we think, decide, and solve problems. This paper presents a series of four experiments on how emotions affect logical reasoning. In two experiments different groups of participants first had to pass a manipulated intelligence test. Their emotional state was altered by giving them feedback, that they performed excellent, poor or on average. Then they completed a set of logical inference problems (with if p, then q statements) either in a Wason selection task paradigm or problems from the logical propositional calculus. Problem content also had either a positive, negative or neutral emotional value. Results showed a clear effect of emotions on reasoning performance. Participants in negative mood performed worse than participants in positive mood, but both groups were outperformed by the neutral mood reasoners. Problem content also had an effect on reasoning performance. In a second set of experiments, participants with exam or spider phobia solved logical problems with contents that were related to their anxiety disorder (spiders or exams). Spider phobic participants' performance was lowered by the spider-content, while exam anxious participants were not affected by the exam-related problem content. Overall, unlike some previous studies, no evidence was found that performance is improved when emotion and content are congruent. These results have consequences for cognitive reasoning research and also for cognitively oriented psychotherapy and the treatment of disorders like depression and anxiety.

## Introduction

In the field of experimental psychology, for a long time the predominant approach was a “divide and conquer” account in which cognition and emotion have been studied in strict isolation (e.g., Ekman and Davidson, 1994; Wilson and Keil, 2001; Holyoak and Morrison, 2005). Yet, in the last decade many researchers have realized that this is a quite artificial distinction and have regarded both systems as distinct but interacting (Dalgleish and Power, 1999; Martin and Clore, 2001). This new line of research resulted in many interesting findings and showed that emotions can have an influence on how we think and how successful we are at solving cognitive tasks (e.g., Schwarz and Clore, 1983; Bless et al., 1996; Schwarz and Skurnik, 2003). Such findings are not only relevant for basic cognitive research, such as reasoning (e.g., Blanchette, 2014), but may also have implications for cognitively oriented psychotherapy and the treatment of disorders like depression and anxiety.

In the present paper we explore the effect of emotion on a cognitive task that is often considered to be a test of rational thinking par excellence: logical reasoning. We start with a brief description of the logical problems that were used in our study. Then we summarize what is currently known about the connection between logical reasoning and emotional states. In the main part of the paper, we describe our hypotheses concerning the connection between logical reasoning and emotional states and then report a series of four experiments, two with a mood induction and two with participants who have a fear of either exams or spiders. In the final section we discuss the connection between logical reasoning and emotions and draw some general conclusions.

### Logical reasoning problems

Logical reasoning goes back to the antique Greek philosopher Aristotle and is today considered to be essential for the success of people in school and daily life and all kinds of scientific discoveries (Johnson-Laird, 2006). In the psychological lab it is often investigated by means of conditional reasoning tasks. Such tasks are composed of a first premise, a second premise and a conclusion. The first premise consists of an “if p, then q” statement that posits q to be true if p is true. The second premise refers to the truth of the antecedent (“if” part) or the consequent (“then” part). The participants‘ task is to decide whether the conclusion follows logically from the two given premises. In this regard, two inferences are valid and two are invalid (given they are interpreted as implications and not as biconditionals, i.e., as “if and only if”). The two valid inferences are modus ponens (MP; “if p, then q, and p is true, then q is true”) and modus tollens (MT; “if p, then q, and q is false, then p is false”), whereas the two invalid inferences are affirmation of consequent (AC; “if p, then q, and q is true, then p is true”) and denial of antecedent (DA; “if p, then q, and p is false, then q is false”). This type of reasoning task was used for Experiments 2–4 while a Wason selection task (Wason, 1966) was used for Experiment 1. The classical Wason selection task (WST) consists of a conditional rule (e.g., “If a card has a vowel on one side, then it has an even number on the other side.”) accompanied by four cards marked with a letter or number, visible only from one side (e.g., A, D, 2, 3). Thus, one side of the card presents the truth or falsity of the antecedent (e.g., A, D) and the other side the truth or falsity of the consequence (e.g., 2, 3). This task requires turning over only those cards which are needed in order to check the validity of the rule. The logically correct response is to turn over the A-card (to check whether the other side is marked with an even number, MP) and the 3-card (because this is not an even number and therefore no vowel should be on the other side, MT). For reasons of brevity, the reader is referred to Johnson-Laird (2006) and Knauff (2007) for a detailed overview of the different types of reasoning problems used in the present paper. We used these tasks in the present work since sentential conditional tasks and the Wason selection task are the best understood problems of logical reasoning research (overview in Johnson-Laird and Byrne, 2002).

### Previous studies and main hypotheses

Several studies on logical reasoning found that participants' performance is modulated by their emotional state. In several experiments, participants underwent a mood induction or were recruited based on their pre-existing emotional state. In both conditions, the emotional state often resulted in a deterioration of reasoning performance (Oaksford et al., 1996). In another study participants were recruited because they reported being depressed (Channon and Baker, 1994). They were presented with categorical syllogisms and their performance was worse than that of non-depressed participants. One possible explanation is that emotionally congruent information (e.g., sad content in case of being depressed) put additional load on working memory (e.g., Baddeley, 2003). Other explanations are that different emotional states affect people's motivation to solve rather complex cognitive tasks (Melton, 1995) or that the emotional state affects how attention is allocated (e.g., Gable and Harmon-Jones, 2012) even with positive material (e.g., Gable and Harmon-Jones, 2013).

The content of the reasoning task can also have an effect on performance. For instance, the content can result in a stereotypical reaction which negatively affects performance on a conditional reasoning task (Lefford, 1946; see also De Jong et al., 1998). Other studies have shown that negative as well as positive content has a detrimental effect on conditional reasoning performance as opposed to neutral content which may be due to reduced working memory resources (Blanchette and Richards, 2004; Blanchette, 2006). The problem content can also be freed from any semantic value by using non-words that have been conditioned via classical conditioning to assume an emotional value. Therefore, the effect of non-semantic emotional material on reasoning performance can be investigated. Classical conditioning has been used to condition non-words and neutral words with a negative or positive emotional value and resulted in participants providing fewer logically valid answers in a conditional reasoning task (Blanchette and Richards, 2004; Blanchette, 2006). The hypothesis that emotions affect how conditional reasoning tasks are interpreted could not be confirmed (Blanchette, 2006).

The literature review shows that mood and emotional problem content negatively affect logical reasoning performance. However, the effects on reasoning performance are still ambiguous, in particular when mood is combined with a problem content that is relevant to the mood, e.g., a participant in a sad mood is presented with a sad reasoning problem about bereavement (mood and content are congruent). Some studies have shown that such a combination results in worse performance. Health-anxiety patients, when reasoning about health-threats in a Wason selection task, have a threat-confirming strategy (Smeets et al., 2000), for example, they very likely interpret a tremor as a sign of Parkinson's disease or chest pain as an indicator for cardiac infarction, etc., even though other—less dangerous—causes are much more likely. Thus, threat-confirming participants select the card that confirms (rather than falsifies) their fears about the anticipated illness. Controls that do not have health-anxiety do not show such a bias when reasoning about health-threats. These findings are similar to another study that also used a Wason selection task where spider-phobic participants confirmed danger rules and falsified safety rules more often for phobia-relevant information than controls (De Jong et al., 1997a). Furthermore, socially anxious participants performed worse in relational inference tasks when the content was relevant to social anxiety as opposed to neutral content (Vroling and de Jong, 2009). However, spider phobic patients compared to non-phobic controls performed worse when the reasoning problem's content was specifically related to their phobia as well as when it contained general threat material (De Jong et al., 1997b).

Other studies found no difference between control participants in a neutral mood, participants with health-anxiety (De Jong et al., 1998) or participants who were not recruited from a clinical population but nevertheless reported anxiety symptoms (Vroling and de Jong, 2010). Participants in a neutral mood as well as anxious participants performed worse in the threat condition. Lastly, some studies even found a beneficial effect of emotions on logical reasoning performance. After the bombing in London in 2005 a study was carried out to investigate if the increased amount of fear which was related to the bombing, has an impact on the performance of participants when solving conditional reasoning tasks that were related in content to the bombing (Blanchette et al., 2007). It resulted that fearful participants provided more correct responses on a reasoning task with fear-related content than participants that did not report a high level of fear. In another study participants that had been primed to be angry or who remembered an incident when they have been cheated on, performed better when the reasoning task involved detecting cheaters (Chang and Wilson, 2004). This mood congruent effect was not found when participants who remembered an altruistic incident had to detect altruists. An evolutionary psychology explanation is offered for these findings as the authors suggest that the ability to detect cheaters provides an evolutionary advantage (Chang and Wilson, 2004).

The ambiguous results in the literature motivated us to bring together the effect of the reasoners' emotional state and the effect of the reasoning problems' emotionally-laden content. Based on this combination we formulated and tested the following hypotheses:

1. Positive and negative emotion^1^ will result in a reduction of reasoning performance.
2. Positive and negative problem content will result in a reduction of reasoning performance.
3. There will be an interaction between the person's emotional state and the emotional content of the problem.

To test these hypotheses, four experiments have been carried out to investigate the effect of emotion, problem content and the combination of the two on reasoning performance. The experiments are:

- Experiment 1: Positive, negative or neutral emotion (induced) paired with a Wason selection task that had positive, negative or neutral problem content.
- Experiment 2: Positive, negative or neutral emotion (induced) paired with conditional reasoning tasks that had positive, negative or neutral problem content.
- Experiment 3: Anxious or neutral emotion (spider-phobic or non-phobic participants) paired with conditional reasoning tasks that had neutral, negative or anxious (phobia-relevant) content.
- Experiment 4: Anxious or neutral emotion (exam anxiety or confidence) paired with conditional reasoning tasks that had neutral, negative or anxious (exam anxiety-relevant) content.

## Experiment 1: emotions in the wason selection task

This experiment was designed in order to test the hypotheses that emotion and emotional content have a disrupting effect on reasoning performance. The participants' emotion was either neutral or induced to be positive or negative and then they had to solve Wason selection tasks. The content of the reasoning tasks which all participants had to solve was positive, negative or neutral as well.

### Methods

#### Participants

Thirty students from the University of Giessen participated in this study (mean age: 22.93 years; range: 19–30 years; 18 female, 12 male). They did not participate in any previous investigations on conditional reasoning and they received a monetary compensation of eight Euro. The participants came from a range of disciplines and none of them were psychology students. They were all native German speakers and provided informed written consent.

#### Design and materials

First, the emotional state of the participants was measured with the German version of the positive and negative affect schedule (PANAS; Watson et al., 1988; Krohne et al., 1996) with which a score for negative and one for positive affect can be computed. Then the participants' emotional state was altered by a manipulated IQ-test. The procedure is described below. However, participants were not told that their emotional state was to be altered with a success-failure-method and they were randomly assigned to the “success group,” “neutral group,” and “failure group.” This method has high reliability and ecological validity (Nummenmaa and Niemi, 2004).

**Figure 1:**
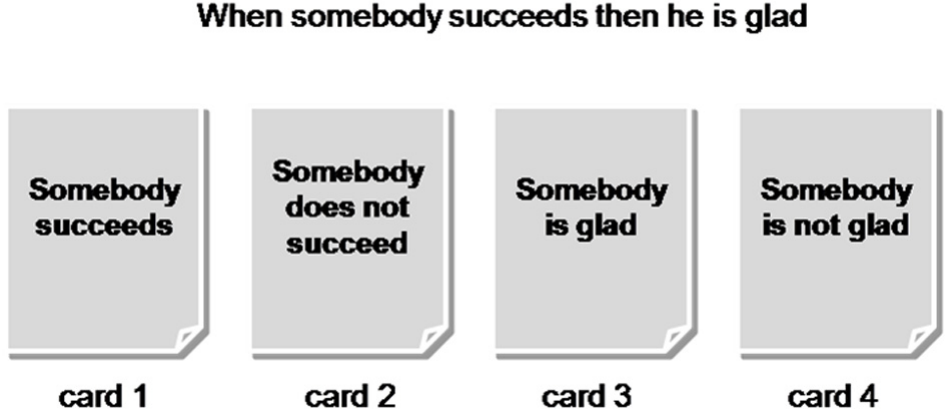
**Example of a WST problem with the four corresponding cards**.

During the logical reasoning task participants had to solve 24 Wason selection tasks based on the three types of content (positive, negative, and neutral). While Wason selection tasks with positive emotional value described success situations, the negative ones described failure situations. This was done to create a link between emotion and the content of the reasoning material. Table 1 shows examples of the positive, negative, and neutral logical reasoning problems. The sentences were presented in German language. Each problem was presented by means of four different virtual cards on a computer screen as can be seen in Figure 1. The participants were told that each card contained one part of the rule on one side and the other part of the rule on the other side. On one set of cards, for example, one side of the card contained the information about whether somebody succeeds or not and on the other side whether somebody is glad or not (the correct answer in our example is card 1 and card 4 which means to verify and to falsify the rule; card 1 and card 3 which is the empirically most frequent answer means that participants in both cases try to verify the rule). The order of cards on the screen was pseudo-randomized and the order of Wason selection problems was completely randomized across participants.

**Table 1:** **Examples for negative (mirroring failure situations), positive (mirroring success situations) and neutral rules (words and sentences were presented in German language in all experiments)**.

**Type of content**	**Example of statement**
Positive	When somebody passes an exam, then he is happy
	When somebody triumphed, then he is lucky
Negative	When somebody feels overstrained, then he is sad
	When somebody has self-doubts, then he is depressed
Neutral	When somebody is cabinet maker, then he works with wood
	When somebody showers, then he uses shampoo

#### Procedure

Participants were tested individually in a quiet laboratory room at the Department of Psychology of the University of Giessen. Prior to the experiment they were informed about the procedure. The emotional state of the participants was measured with the German version of the positive and negative affect schedule (PANAS; Watson et al., 1988; Krohne et al., 1996) with which a score for negative and for positive affect can be computed. This scale is based on 10 positive and 10 negative adjectives. Participants are required to state the emotional intensity of each word on a five point scale: 1 = “not at all,” 2 = “a little,” 3 = “moderately,” 4 = “quite a bit,” and 5 = “very much.” Thus, for the positive as well as for the negative affect a score ranging between 10 and 50 points could be computed. Examples of the test adjectives are: afraid, guilty, inspired, proud, etc. This emotion measurement schedule has been validated in several studies (e.g., Krohne et al., 1996; Crawford and Henry, 2004).

After that the participants carried out a subset of items from the IST2000R (Amthauer et al., 2001), which is a popular IQ-test in psychological research and practice. This subtest consisted of 13 items from three different categories: sentence completion, calculation and matrix tasks. These items were selected from all items by using the norming data from the intelligence test. For one group we selected the 13 problems that are most difficult, for the second group we selected items with moderate difficulty according to the norms and for the third group we used the easiest items from the IST2000R. Here is one example for the calculation tasks: (24/144) × 96 = ? (difficult), (3/6) + (20/8) = ? (moderate), and 8 × 123 = ? (easy). The items were presented on a sheet of paper and had to be solved by the participants in a limited time. In order to boost the effect of the emotion manipulation we also told them that the test was especially developed to predict academic success and that an average student solves approximately 50% of the items correctly. The time limit was 15 min. After finishing the test the participants received a manipulated verbal feedback on their performance to influence their emotional state. The feedback for the negative emotion group with the difficult problems was: “We are sorry to say that the analysis of your data showed that your performance was below the average student performance.” The feedback for the neutral emotion group with moderate item difficulty was: “The analysis of your data showed that your performance was on average student performance.” The participants from the positive emotion group with the easy items were told that their performance was above the average of student performance. Please note that this feedback did not reflect their real performance, because even if participants managed to solve the difficult problems they got the negative feedback. Accordingly, the participants in the positive emotional group got positive feedback even if they failed to solve the problems.

After this the emotional state was assessed again to see whether the mood induction was successful. Finally they were given the Wason selection tasks. In order to hide the real purpose of our study, we told the participants they had to do the PANAS since current emotions could influence their performance on intelligence tests and that we wanted to control for this. All our experiments were approved by the ethics committee of the German Psychological Association (DGPs).

The experiment then started with the emotion induction sequence [PANAS (t1), intelligence test items, feedback and PANAS(t2)], followed by the 24 Wason selection tasks. A computer administered the Wason selection problems using the SuperLab 4.0 software (Cedrus Corporation, San Pedro, CA) and recorded participants‘ answers (in all experiments). A self-paced design was used for data collection. When the problem with the four cards was presented on the screen participants had to decide which of the cards they would like to turn over in order to check the validity of the given rule. They were asked to pick one or more cards by pressing the corresponding keys on the labeled keyboard. For instance, to turn over card 1 they had to press the “1” key, which was clearly labeled “card 1.” The problems were separated by the instruction to press the <spacebar> whenever ready for the next problem. At the beginning one practice problem was presented to familiarize participants with the task but no feedback was given. At the end of the experiment all participants were informed about the true nature, the intention and the manipulations of the experiment. In all experiments data was analyzed with SPSS19 (IBM^©^) using analyses of variance (ANOVAs) and *t*-tests (details are given in each of the Results sections).

### Results

**Figure 2:**
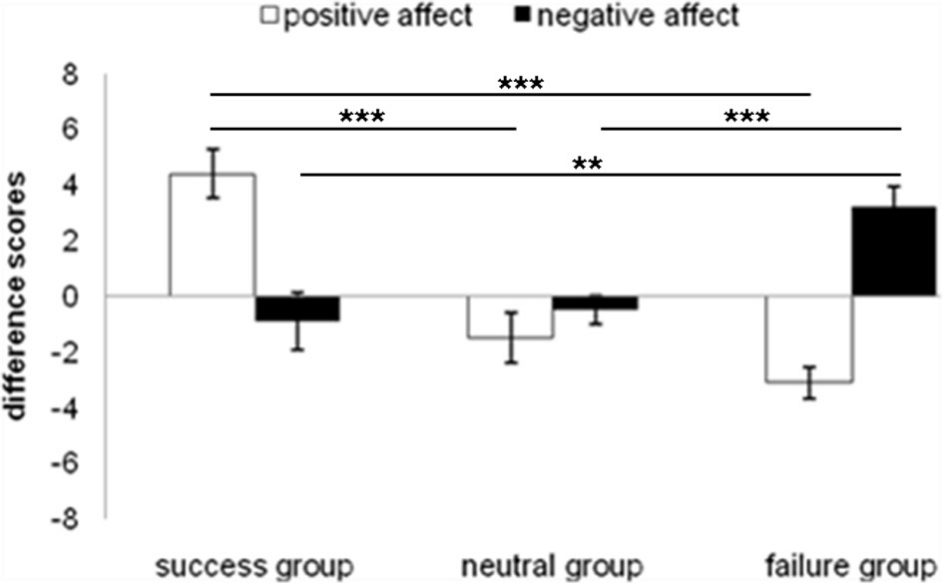
**Difference scores (t_2_ - t_1_) for the emotion induction of Experiment 1 for each group**. ^**^p ≤ 0.01, ^***^p ≤ 0.001.

The emotion manipulation was successful, as can be seen in Figure 2. The success group revealed a significant increase of positive affect from t1 to t2 [*t*_(9)_ = −4.906, *p* = 0.001], while the negative affect decreased. The failure group scores showed a significant decrease in positive affect [*t*_(9)_ = 5.471, p < 0.001] and a significant increase in negative affect [*t*_(9)_ = −4.226, *p* > 0.01]. For the neutral group no differences were found, neither for the positive nor the negative affect. A One-Way ANOVA including the factor “positive difference scores” and a between-subject factor (neutral, success, or failure group) revealed significant group differences [*F*_(2, 27)_ = 23.964, *Mean Squared Error* (*MSE*) = 6.511, *p* < 0.001]. A second One-Way ANOVA with the factor “negative difference scores” also showed group differences [*F*_(2, 27)_ = 7.975, *MSE* = 6.407, *p* < 0.01]. Planned *t*-tests for independent samples revealed significant differences in positive difference scores for the success and neutral group [*t*_(18)_ = 4.618, *p* < 0.001] and for the success and failure group [*t*_(18)_ = 7.069, *p* < 0.001]. Significant differences in the negative scores were observed for the comparison between success group and failure group [*t*_(18)_ = −3.192, *p* < 0.01], as well as for the comparison between failure group and neutral group [*t*_(18)_ = 4.024, *p* = 0.001].

**Figure 3:**
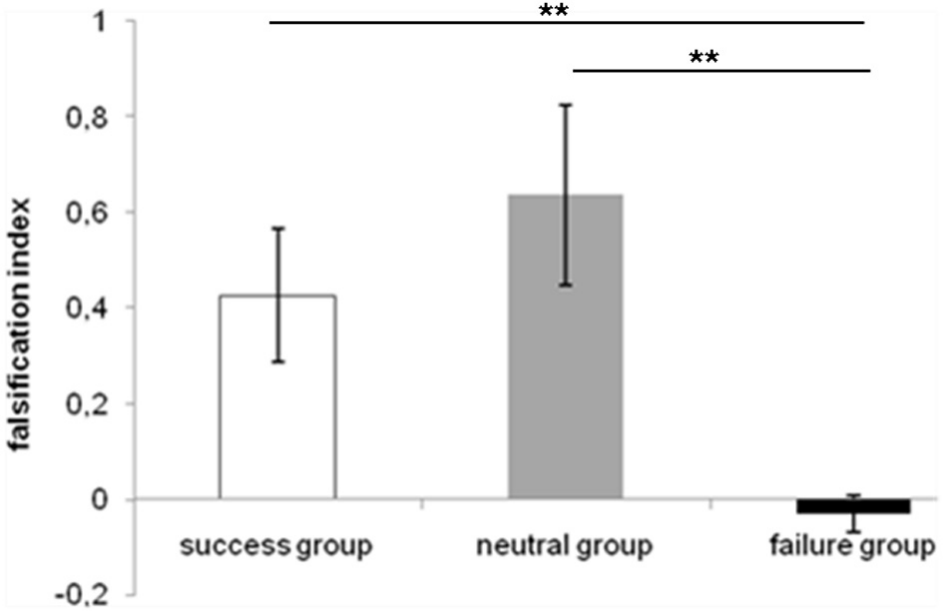
**Falsification index (ranging from −2 to 2) for the WSTs for each group**. It represents the choices of p *and* not-q in order to falsify the rule (modus tollens). ^**^p ≤ 0.01.

On average, participants only solved 5% of the problems correctly (turning card 1 and card 4; every other decision was incorrect which occurred in 95% of the cases). We are aware that this performance is very low. Therefore, we initially thought that it might be useful to statistically test whether this performance significantly differs from chance level. We then decided, however, not to follow this idea, because our results agree with the entire literature on the Wason selection task (Wason and Johnson-Laird, 1972; overview in Manktelow, 2004). Moreover, the usual way of dealing with these low performance rates is to use the “falsification index” and the “confirmation index,” which have been introduced by Oaksford et al. (1996). These indices give a better performance measurement than just comparing correct answers in the Wason selection tasks. The indices can range from +2 to −2 and provide a measure of whether an individual tried to verify or to falsify a given rule by turning over certain cards or card combinations (Oaksford et al., 1996; Chang and Wilson, 2004). The falsification index (FI) is computed with the formula FI = (p + not q) − (not p + q) and stands for the participants' tendency to choose the p and not q cards in order to falsify the rule. Note, that a score of +2 is equivalent to full logicality. The confirmation index (CI) is the “complement” of the falsification index; it stands for the degree to which participants choose the p and q cards in order to confirm the rule. It is calculated with the formula CI = (p + q) − (not p + not q) (Oaksford et al., 1996). Note that a score of +2 is equivalent to a confirming strategy without falsifying the given rule. The mean falsification index is shown in Figure 3.

**Figure 4:**
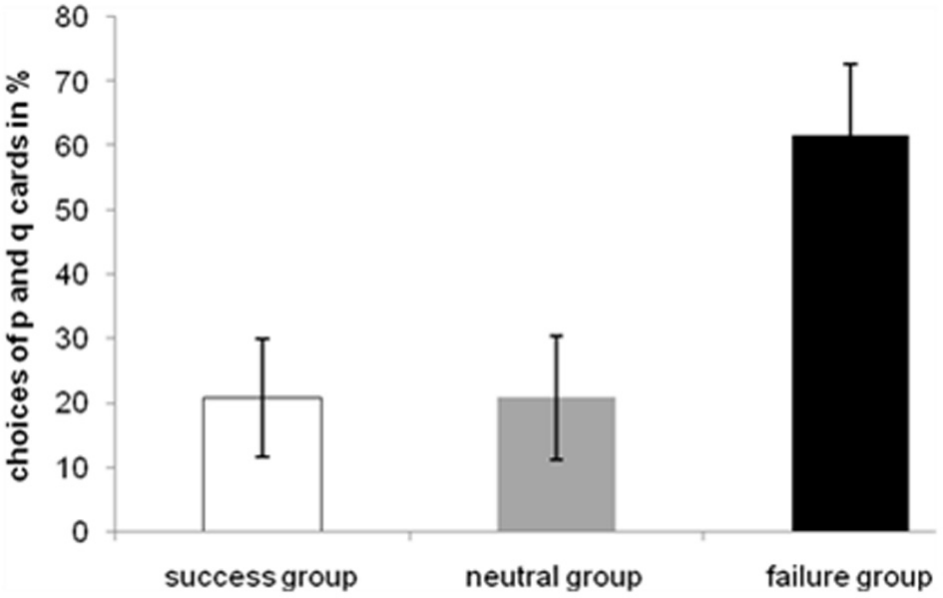
**Choices of the p and q cards of the WSTs in relative frequencies (%) for each group**. With p *and* q (modus ponens) participants tried to confirm the rule.

Falsification indices were then used in an ANOVA including the within-subject factor content (positive, negative, neutral) and the between-subject factor group (success, failure, neutral). This analysis showed that emotion of participants resulted in a significant difference, *F*_(2, 27)_ = 6.033, *MSE* = 0.574, *p* < 0.01, but not the content of the reasoning problem. *Post-hoc t*-tests showed that the falsification index of the failure group differed significantly from those of the neutral group [*t*_(3.737)_ = −3.435, *p* < 0.01] and the success group [*t*_(10.353)_ = 3.14, *p* = 0.01]. Overall, the neutral group [*Mean Falisification Index (MFI)* = 0.636, *Standard Error (SE)* = 0.19] performed better than the success group (*MFI* = 0.426, *SE* = 0.14) and the success group in turn was better than the failure group (*MFI* = − 0.029, *SE* = 0.038). A more detailed descriptive analysis showed that this effect is due to a specific type of error. In fact, participants in the failure group have chosen the p *and* q card most frequently (Figure 4).

### Discussion

The results indicate that the emotions of an individual have an effect on reasoning performance independent from task content. In particular, a negative emotion resulted in a lower falsification index meaning that participants in a negative emotional state were more likely to deviate from logical norms. The participants in a positive state were also not as good as the neutral group, but this difference was less pronounced. Overall, participants in a neutral emotional state performed best. However, no interaction has been found between participants' emotion and the emotional task content, neither for the falsification index, nor for the confirmation index. Thus, it was not easier for individuals in positive (negative) emotion to solve Wason selection tasks with positive (negative) content. The reason for this might be that the Wason selection task overall is too difficult to solve and that there is no generally accepted theory about what makes the tasks so complex. A recent overview of such approaches can be found in Klauer et al. (2007). For our studies the reasons for the difficulty of the Wason selection task are not particularly essential. However, a detrimental result might be that participants' low performance could result in a “floor effect” and thus existing effects of the emotional content might not be visible in the data. In order to control for this possible deficit, a paradigm for the subsequent experiment has been chosen which is known to result in better performance.

## Experiment 2: emotions and conditional reasoning tasks

The intention of this experiment was to use a reasoning task which participants find easier to solve than a Wason selection task. We therefore used a conditional reasoning paradigm. If such a task is easier, any difference between groups' performance should be much clearer and such differences can be more readily attributed to the experimental manipulation. Again, the conditional reasoning tasks had a positive, negative or neutral content and like in the previous experiment, participants' emotions were either induced (positive or negative) or neutral.

### Methods

#### Participants

Thirty students from the University of Giessen participated in this study (mean age: 22.6 years; range: 20–27 years; 22 female, 8 male). They did not participate in any of the other investigations. They received an eight Euro compensation for participation. All participants were naïve with respect to the aim of the study, none were psychology students. All were native German speakers and provided informed written consent.

#### Design and materials

The same success-failure-method which was used in the previous experiment was used for the emotion induction. Reasoning problems consisted of pairs of premises that were followed by a to-be-validated conclusion. Four premise-pairs had a positive, four a neutral and four a negative content. These 12 problems were combined with the four possible inferences: modus ponens (MP), modus tollens (MT), denial of antecedent (DA) and affirmation of consequence (AC), resulting in 48 conditional inferences per participant. All problems were randomized for each participant. Half of the presented conclusions were valid; the other half were invalid. Here are two examples of inferences with a valid conclusion:

Modus ponens/positive emotional content
Premise 1: When a person succeeds, then the person is glad.
Premise 2: A person succeeds.
Conclusion: This person is glad.
Modus tollens/negative emotional content
Premise 1: When a person performs poorly, then this person is angry.
Premise 2: A person is not angry.
Conclusion: This person did not perform poorly.

#### Procedure

The participants were tested individually in a quiet laboratory room at the Department of Psychology of the University of Giessen. Prior to the experiment, the participants were again instructed about the procedure of the experiment. Subsequently, the emotion induction started and resulted in a “success group,” a “failure group,” and a “neutral group.” Then the inferences were presented on a computer screen. A self-paced design was used. After reading the first premise on the screen, participants had to press the space bar to reach the next premise, then again the space bar to reach the conclusion. While both premises were presented in black letters the conclusion was presented in red. The task required an evaluation whether the conclusion followed necessarily from the premises (no evaluations as biconditionals). Participants responded by pressing either a “Yes” key or a “No” key on the keyboard. There were two practice trials at the beginning of the experiment but no feedback was given. At the end of the experiment there was a debriefing and a detailed explanation of the true purpose of the experiment.

### Results

The emotion induction was again successful. In the success group the positive affect was elevated and the negative affect reduced (similar to the previous experiments' mood induction). In the failure group the positive affect decreased and the negative affect increased (although the latter was not significant, due to a large standard error). No alteration for positive and negative affect was found in the neutral group. The ANOVA revealed significant group differences [*F*_(2, 27)_ = 15.964, *MSE* = 13.607, *p* < 0.001] and the *t*-tests for independent samples showed that the success and neutral group were dissimilar in the difference scores of positive affect [*t*_(18)_ = 2.146, *p* < 0.05], as well as the success and failure group [*t*_(18)_ = 5.666, *p* < 0.001] and the failure and neutral group [*t*_(18)_ = −3.854, *p* < 0.01].

Performance for the sentential conditional inference problems was better than for the Wason selection tasks as 61.46% of the problems were correctly solved. Error rates were compared using an ANOVA for the emotionality of the participants (success, failure, and neutral group) and the emotional content (positive, negative, neutral). Significant differences were found for both factors.

**Figure 5:**
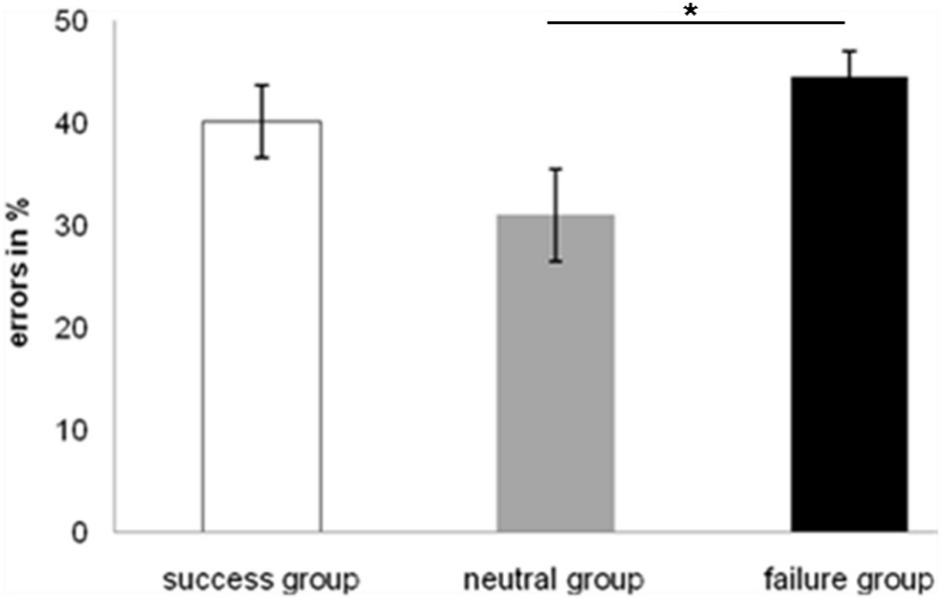
**Error rates in relative frequencies (%) for the conditional reasoning task for each group**. ^*^p ≤ 0.05.

With respect to the emotional state the performance of the participants in the three groups was reliably different [*F*_(2, 27)_ = 3.68, *MSE* = 2.492, *p* < 0.05] and paired sample *t*-tests show that error rates for the failure group were significantly higher compared to the neutral group [*t*_(18)_ = 2.622, *p* < 0.05]. The neutral group showed best performance [*Mean (M)* = 0.310, *SE* = 0.046] followed by the success group (*M* = 0.402, *SE* = 0.035) and the failure group which committed most errors (*M* = 0.446, *SE* = 0.024). These results are represented in Figure 5.

**Figure 6:**
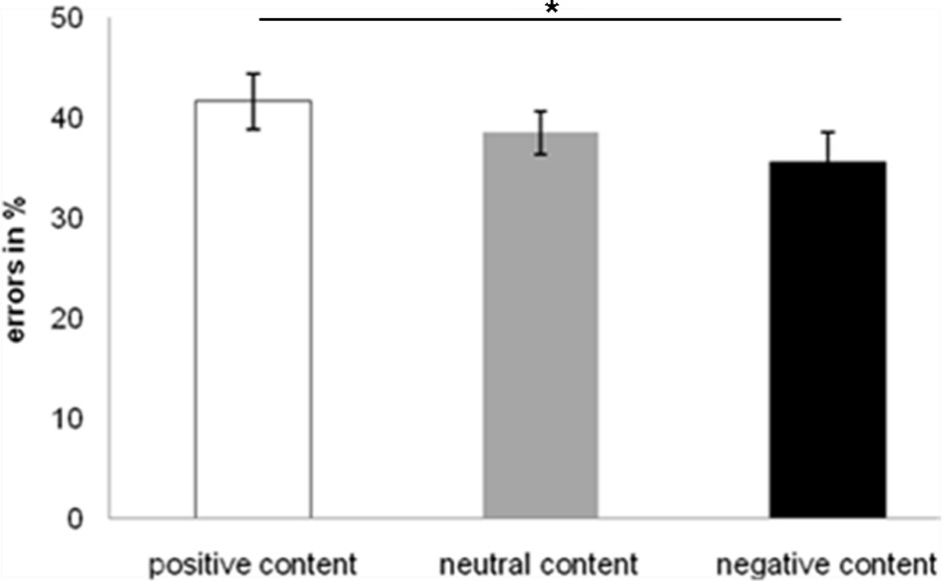
**Error rates in relative frequencies (%) for the conditional reasoning task for each type of content**. ^*^p ≤ 0.05.

The difference between positive and negative content of the reasoning problems was also significant. The ANOVA showed a significant main effect [*F*_(2, 54)_ = 3.159, *MSE* = 0.555, *p* = 0.05] and the *post-hoc* paired sample *t*-tests revealed a significant difference in error rates between positive and negative content [*t*_(29)_ = 2.491, *p* < 0.05]. The fewest errors were made with negative content (*M* = 0.356, *SE* = 0.029), followed by neutral content (*M* = 0.385, *SE* = 0.022), and positive content (*M* = 0.417, *SE* = 0.028). This is visualized in Figure 6. However, no interaction was found between emotional state and task content.

### Discussion

The reported findings show that several factors can influence reasoning performance. Performance can be affected either by the emotion of the individual or the content of the problem or the type of inference.

The effect of emotion might be due to the fact that emotion results in representations in working memory that occupy the same subsystems that are also needed for reasoning (Oaksford et al., 1996). The content effect is also interesting since it challenges previous findings. While we found fewer errors in inferences with negative content, Blanchette and Richards (2004) found that emotions impair reasoning performance no matter whether they are positive or negative.

## Experiment 3: spider-phobic participants and conditional reasoning

In contrast to the previous experiments the sample for this experiment was selected from a population with spider phobia. Therefore, it was not necessary to induce emotions as participants were selected for their anxiety with high ecological validity. This was done to expand the findings of the previous experiments in order to see if a difference in performance can be found for participants that already have pre-existing moods in certain situations without any mood induction. Additionally, we were interested in whether content relevant to the illness of such participants has any effect on their reasoning abilities.

### Methods

#### Participants

Nine spider phobic students (mean age: 22.33 years; range: 20–26 years; 7 female, 2 male) and seven non-phobic control students (mean age: 22.86 years; range: 20–26 years; 7 female) from the University of Giessen participated in the experiment. Participants were selected from a larger sample by means of scores on the Spider Phobia Questionnaire (SPQ; Klorman et al., 1974). SPQ scores of spider fearful students (*M* = 20.22; *SE* = 0.878) were significantly higher than those of the non-fearful control students (*M* = 2.00; *SE* = 0.873) [*t*_(14)_ = −14.459; *p* < 0.001]. Each participant received five Euro or a course credit for participation. Moreover, we controlled for participants being no psychology students (thus, no pre-experience with logical reasoning tasks) and all were native German speakers. All participants provided informed written consent.

#### Design and materials

Design and procedure were similar to that of Experiment 2. Forty-eight reasoning problems consisted of pairs of premises that were followed by a to-be-validated conclusion. However, the content differed because four statements had a spider phobia relevant content, four were generally negative and four neutral. The presentation of the 48 three-term problems was randomized across participants. Examples of the statements are presented in Table 2.

**Table 2:** **Examples of statements with different content**.

**Type of content**	**Example of statement**
Spider phobia relevant	When a person sees a toy spider, then the person is scared witless
Negative	When a person is anorexic, then the person has to be force-fed
Neutral	When a person is a craftsman, then the person has served an apprenticeship

#### Procedure

All participants were tested individually in a quiet room at the Department of Psychology of the University of Giessen. At the beginning participants filled out the SPQ. Afterwards the logical reasoning tasks had to be solved. Presentation of problems and recording of responses was identical to Experiment 2.

### Results

Error rates of the conditional reasoning task were compared using an ANOVA with the between-subject factor group and the two within-subject factors content and type of reasoning.

**Figure 7:**
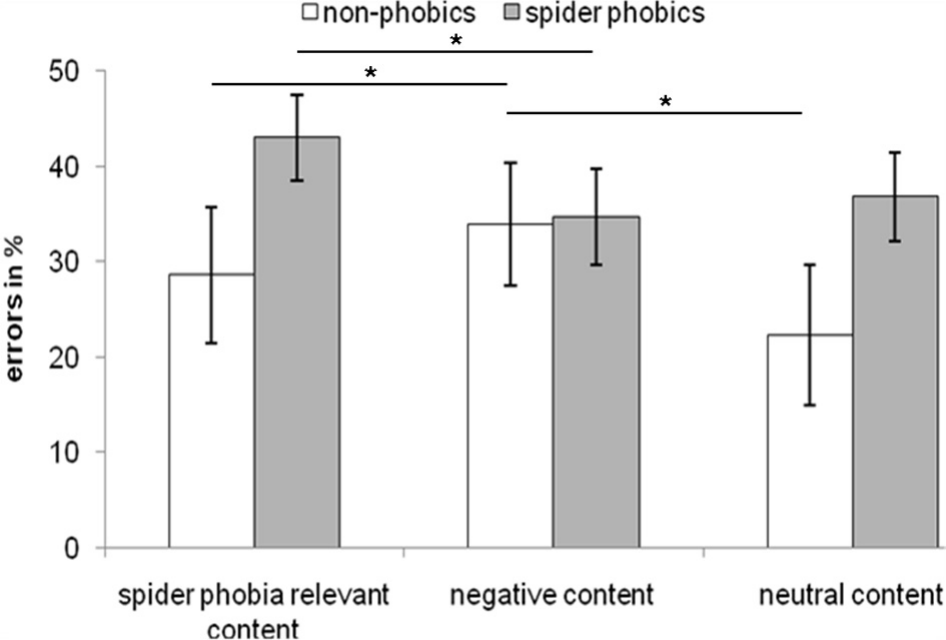
**Error rates in relative frequencies (%) for the spider phobic and non-phobic participants**. ^*^p ≤ 0.05.

For the content of reasoning problems a significant main effect was obtained [*F*_(2, 28)_ = 4.645; *p* < 0.05]. Further paired *t*-tests showed that error rates for spider phobia relevant problems (*M* = 36.72%; *SE* = 4.30%) resulted in significantly more errors than neutral ones (*M* = 30.47%; *SE* = 4.41%) [*t*_(15)_ =2.928; *p* = 0.01]. This was due to spider phobics performing worse on phobia relevant contents. This interaction between problem content and emotion was significantly different [*F*_(2, 28)_ = 6.807; *p* < 0.01]. A *post-hoc* paired *t*-test revealed that spider phobics performed significantly worse for inference problems with spider phobia relevant content (*M* = 43.06%; *SE* = 4.47%) compared to negative ones (*M* = 34.72%; *SE* = 5.01%) [*t*_(8)_ = 2.667; *p* < 0.05]. Furthermore, phobia relevant problems resulted in more errors than neutral ones (*M* = 36.81%; *SE* = 4.71%) but marginally failed to reach significance [*t*_(8)_ = 2.268; *p* = 0.053]. However, non-phobics made significantly more errors for inferences with negative content (*M* = 33.93%; *SE* = 6.38%) compared to spider phobia relevant (*M* = 28.57%; *SE* = 7.20%) [*t*_(6)_ = −2.521; *p* < 0.05] and neutral problems (*M* = 22.32%; *SE* = 7.33%) [*t*_(6)_ = −3.653; *p* < 0.05]. This interaction pattern between the groups and the task content of the conditional reasoning task is visualized in Figure 7.

### Discussion

Our results show that Spider phobics' performance was worst on problems related to spider phobia. We are aware of the fact that our sample size is rather small. One reason was that it is difficult to find spider phobics, because they usually avoid situations where they are confronted with spiders. However, our control group was also small. The reason for that is that we initially also tested nine participants in the control group (same number as in the experimental group) but then we had to eliminate two participants (due to response strategy, incomplete data recording) and could not replace them by two new participants for technical reasons. However, we do not think that this is a serious problem, because even with this small sample size our differences reached the level of statistical significance. Given these thoughts we think that our results reliably show that illness related tasks impair reasoning for anxiety patients.

There are a couple of possible explanations of how (positive and negative) emotions impeded on reasoning performance. One explanation is that all kinds of emotions have negative effects on the motivation or effort of the participants (e.g., Lefford, 1946). Other explanations are based on dual process models (System or Type 1: automatic, fast, intuitive, based on prior knowledge; System or Type 2: effortful, slow, explicit, rule-based, e.g., Stanovich, 2010). A good overview on the different theories is provided in Blanchette (2014). However, we believe that the most reasonable explanation for the current findings is provided by the suppression theory (Oaksford et al., 1996): processing phobia relevant material comprised the confrontation with the phobic object which causes fear. This yields a strong emotional response resulting in a pre-load of working memory resources. Moreover, there is evidence that spider phobia could change reasoning patterns. De Jong et al. (1997a) showed that spider phobics tend to rely on a danger-confirming reasoning strategy while solving phobia relevant Wason selection tasks. While spider phobics performed worst on phobia relevant problems in our study, non-phobics revealed worst performance on problems with negative content. These results are in line with Blanchette and Richards (2004) and Blanchette (2006). Overall affirmation of consequence and denial of antecedent with spider phobia relevant and negative content resulted in more errors which is similar to findings of Blanchette and Richards (2004).

## Experiment 4: exam-anxious participants and conditional reasoning tasks

This experiment was designed to investigate if the effect found in Experiment 3 extends to other anxiety related conditions such as exam-anxiety. Therefore, participants were also selected based on their anxious state and some of the problems had an emotional content which was relevant to exam-anxiety while others were neutral or generally negative.

### Methods

#### Participants

The sample consisted of 17 students with exam anxiety and 17 students without exam-anxiety. They have been selected from a larger sample (*N* = 47) based on their scores of a measure for exam-anxiety (Hodapp, 1991). They were all female because exam-anxiety is more prevalent amongst women (Zeidner and Safir, 1989; Chapell et al., 2005; Wacker et al., 2008). The age range was 20–29 years (mean age for participants with exam-anxiety: 24.24 years, without exam-anxiety: 23.12 years). For remuneration they could choose to receive five Euro or a course credit. Psychology students and people who have already taken part in experiments about this topic were excluded. All participants were native German speakers and provided informed written consent.

#### Design and materials

Participants were assessed with the TAI-G (Hodapp, 1991), a measure for exam-anxiety, in order to differentiate between exam-anxious and non-anxious participants. The TAI-G consists of 30 statements which describe emotions and thoughts in exam situations. Participants are asked how well those statements describe them when they have to take exams. Statements were ranked on a scale from “never” (1), “sometimes” (2), “often” (3) to “almost always” (4).

Examples of such statements are:

“I have a strange sensation in my stomach.”

“Thoughts suddenly start racing through my head that block me.”

“I worry that something could go wrong.”

Scores of the TAI-G range from 30 to 120. In order to be classified as exam-anxious a minimum score of 84 is necessary while a score below 54 is classified as non-exam-anxious. Those limits were obtained in a study with 730 students (Wacker et al., 2008) in which one standard deviation (*SD* = 14.8) was subtracted from the mean score (*m* = 69.1) to obtain the lower limit and added to obtain the upper limit.

Once participants finished the TAI-G, they were given the conditional inference problems. The 48 conditional inference problems consisted of “if, then”-statements of which one third were exam-anxiety-related, one third generally negative and one third emotionally neutral. Examples are given in Table 3. Presentation of the problems and recording of answers was identical to Experiments 2 and 3.

**Table 3:** **Examples of statements with different content**.

**Type of content**	**Example of statement**
Exam-related	If a person is waiting in front of the exam room, then the person is nervous
Negative	If a person has breast cancer, then the person has lumps in her breasts
Neutral	If a person is thirteen years old, then the person is still a child

### Results

The selection of exam-phobic and non-exam phobic groups of participants was successful. The group of the exam-anxious participants had a TAI-G score that ranged from 84 to 107 and a mean of 97 (*SE* = 1.586). The group of non-exam-anxious participants had a score between 39 to 54 and a mean of 48 (*SE* = 1.047). A *t*-test for independent samples showed a significant difference between groups [*t*_(32)_ = 25.788, *p* < 0.001].

Moreover, as expected, the ANOVA revealed a significant main effect with respect to content [*F*_(2, 64)_ = 8.058; *p* = 0.001]. *Post-hoc t*-tests showed that conditional inference problems with fear-related content (*M* = 44.67%; *SE* = 2.52%) resulted in more errors than other negative (*M* = 36.58%; *SE* = 2.53%) [*t*_(33)_ = 3.703; *p* = 0.001] and neutral problems (*M* = 37.87%; *SE* = 2.80%) [*t*_(33)_ = 2.626; *p* < 0.05]. A repeated measures ANOVA was carried out based on error rates for type of inference (MP, MT, AC, and DA), content (fear-related, negative, and neutral) and exam-anxiety. However, no significant interaction was found for content and group. This means that both exam-anxious and non-exam-anxious participants performed similar across fear-relevant, negative, and neutral problems.

### Discussion

Our results show that exam-anxious and non-exam-anxious participants performed similar across fear-relevant, negative and neutral problems. Inferences about exam-anxiety resulted in reduced performance in both groups. This may be because all participants were currently enrolled at university and so can relate to exam-anxiety. Moreover, physiological changes have been observed in people who are high-exam-anxious as well as low-exam-anxious (Holroyd et al., 1978). Therefore, associations to exam-situations can get triggered which reduce working memory resources and subsequently performance on reasoning problems (Oaksford et al., 1996; Blanchette and Richards, 2004). In contrast to previous findings (Lefford, 1946; De Jong et al., 1998; Blanchette and Richards, 2004; Blanchette, 2006) negative problems did not result in a reduction of performance. Even though these problems were emotional and negative (e.g., “if a person has a miscarriage, then this person will get depressed”) participants may not have been able to relate to the content as it was not as personally relevant to students as the exam-related content.

## General discussion

We conducted two experiments with participants who underwent a mood induction and two with participants that were either anxious about spiders or exams. Experiment 1 showed that the emotions of an individual have an effect on reasoning performance independent from task content. In Experiment 2, we found that reasoning performance can be affected either by the emotion of the individual or the content of the problem or the type of inference. In Experiment 3, spider-phobic participants showed lower reasoning performance in spider-related inferences, but in Experiment 4, exam-anxious participants did not perform worse on inferences with an exam-related content.

The results agree with some of our hypotheses but not with all of our initial assumptions. Our first hypothesis was that positive and negative emotion will result in a reduction of logical reasoning performance. This was confirmed as in the first and second experiment participants in a neutral emotional state outperformed those in negative or positive emotion independent of the task (WST and conditionals). These findings are consistent with previous research (Channon and Baker, 1994; Melton, 1995; Oaksford et al., 1996). When a negative or positive emotional state has been induced in participants this results in a deterioration of performance on a Wason selection task compared to participants in a neutral emotional state (Oaksford et al., 1996). In another study participants were recruited because they reported being depressed (Channon and Baker, 1994). They were presented with categorical syllogisms and their performance was worse than that of non-depressed participants. An explanation that has been offered is that as emotionally congruent information gets retrieved and processed this takes away resources from working memory (e.g., Baddeley, 2003) that should have been used to process the reasoning task. In addition, positive emotional states also result in poorer performance (Melton, 1995), as it is assumed that people in a positive mood pursuit more global reasoning strategies, paying less attention, and are therefore more prone to errors than people in a negative, analytic mood.

Our results concerning the second hypothesis (predicting a detrimental effect on performance of positive and negative problem content) are mixed. It was confirmed by the third experiment in which non-phobic participants performed best when the content was neutral. On the other hand, the content had no effect on performance in the first experiment, and in the second experiment, best performance was measured with negative content, whereas most errors were committed with positive content. In the fourth experiment there was no difference between negative and neutral content and performance was worst with exam-anxiety related content. These findings partially agree with previous research showing that performance is affected when the content is related to general threats because then participants tend to select threat-confirming and safety-falsification strategies in a Wason selection task (De Jong et al., 1998). Other studies have shown that negative as well as positive content has a detrimental effect on conditional reasoning performance as opposed to neutral content which may be due to reduced working memory resources (Blanchette and Richards, 2004; Blanchette, 2006). Furthermore, if the content is controversial, it can stir up emotions that result in a stereotypical reaction that negatively affects performance of a conditional reasoning task (Lefford, 1946). In this study participants made more errors when the content was controversial (e.g., stereotypical responses such as “homeless person are lazy”) as opposed to neutral.

The third hypothesis stating there may be an effect on performance when positive and negative mood is combined with positive and negative problem content was only supported by Experiment 3, which found the expected interaction. Nonetheless, the absence of the suggested interaction in three of four experiments is in line with some previous findings (e.g., De Jong et al., 1998, health-anxiety; Vroling and de Jong, 2010, anxiety symptoms in a non-clinical population).

Only in the third experiment participants who are afraid of spiders performed worse on problems with a spider phobia relevant content compared to a negative content which strengthens other findings (De Jong et al., 1997a,b; Smeets et al., 2000; Vroling and de Jong, 2009). A similar trend was observed for the performance on spider phobia relevant problems compared to neutral ones. Yet this difference was insignificant, maybe a bigger sample would have yielded clearer results. A previous study showed that, when reasoning about health-threats in a Wason selection task, health-anxiety patients have a threat-confirming strategy (Smeets et al., 2000). Controls that do not have health-anxiety do not show such a bias when reasoning about health-threats. These findings are similar to another study that also used a Wason selection task where spider-phobic participants confirmed danger rules and falsified safety rules more often for phobia-relevant information than controls (De Jong et al., 1997a). Furthermore, socially anxious participants performed worse in relational inference tasks when the content was relevant to social anxiety as opposed to neutral content (Vroling and de Jong, 2009). However, spider phobic patients compared to non-phobic controls performed worse when the content of the reasoning problem was specifically related to their phobia as well as when it contained general threat material (De Jong et al., 1997b).

Why did we find no evidence showing that performance is improved when emotion and content are congruent? In Blanchette et al. (2007) fearful participants provided more correct responses on a reasoning task with fear-related content than participants that did not report a high level of fear. In another study participants who had been primed to be angry or who remembered an incident when they had been cheated on performed better when the reasoning task involved detecting cheaters (Chang and Wilson, 2004).

We think that the ambiguity in previous findings (Channon and Baker, 1994; Melton, 1995; Oaksford et al., 1996; Chang and Wilson, 2004; Blanchette et al., 2007) and our own experiments may be due to the differences between samples. The first two experiments induced emotions in participants who were primarily sad and frustrated whereas the last two experiments' participants were anxious. Hence one is not comparing like with like. The latter two experiments can be further differentiated as the third experiment selected people for the control group who are not afraid of spiders. However, most students experience some form of exam-anxiety and the sample of the fourth experiment was entirely made up of students. This may explain why participants who reported exam anxiety as well as those who reported none both performed poorly when the content was exam anxiety related.

According to the suppression theory (Oaksford et al., 1996), emotion has a detrimental effect on performance because resources are otherwise allocated and not available to solve the task at hand. This means that emotional participants should perform worse than those in a neutral state. This has been confirmed in Experiments 1 and 2. Content may give rise to emotion and so similar results due to reduced working memory resources should also be found in experiments with emotional content. In Experiment 3 best performance was with neutral content, possibly because spider-related content triggered a response that used resources of working memory that would otherwise have been used to solve the task (e.g., avoidance strategy). Anxious content in Experiment 4 resulted in worst performance possibly for the same reason.

Thus far we focused on working memory resources, but it is also possible that attentional processes are of major relevance in this context. For example, correct decisions and decision times may be compromised during emotional (especially negative) processing, since emotional processing (in addition to reasoning) requires attentional resources (see for instance the work of Harmon-Jones et al.). However, we cannot fully dissolve this problem of working memory vs. attention at this stage with these experiments.

The findings of Experiment 3 are in contrast to those of Experiments 1 and 2, where no content and interaction effect were found. People with a phobia may perform worse on problems that have a content which is related to their phobia because they try to avoid stimuli that are anxiety-provoking (American Psychiatric Association, 2000). This avoidance is not necessarily found in depressed participants as they tend to ruminate on depressive material (American Psychiatric Association, 2000). While participants in Experiments 1 and 2 were not clinically depressed, the emotion that was induced had a depressive quality and therefore may explain why no interaction was found in these experiments. In addition maybe only anxiogenic stimuli have a depleting effect on working memory and previous research was largely based on anxiety (De Jong et al., 1998; Blanchette and Richards, 2004; Blanchette, 2006). In contrast, Lefford's (1946) material was not anxiogenic but he found an effect. He argued that this was due to a stereotypical response. However, if people do not relate to the content, then this will not result in a stereotypical response.

The reason for why no effect was found in Experiments 1 and 2 might be that the material was not as personally relevant and therefore did not trigger sufficient emotions for an effect to show. This does not explain why in Experiments 2 and 4 best performance was with negative content. One could argue that since this content is negative, participants are more deliberate in order to avoid negative consequences (if personally relevant for them). Furthermore, a more analytic processing style has been proposed for depression (Edwards and Weary, 1993) so that this content may have triggered such a processing style compared to a more global processing strategy with a positive emotion. Considering this one would have expected superior performance for negative emotion in Experiments 1 and 2 which was not the case.

Therefore, more clarity might be achieved if experiments compare personally relevant emotional content and emotional content that is not personally relevant. Content should also be differentiated according to it being anxious or depressive. Furthermore, anxious participants should be compared to depressed participants. A distinction has to be made between avoidance caused by anxiety and rumination caused by depression. If a detrimental effect on performance is found in both groups it has to be investigated whether this has the same cause, namely depleted working memory resources (or attentional resources).

From a psychotherapeutic point of view our studies are interesting as they show that spider phobic patients do not only show inadequate emotional responses to spiders. They, in fact, also show a decrement in performing cognitive tasks, such as logical reasoning if they have to do with spiders. The study shows an apparent connection between reported fear on the SPQ (Klorman et al., 1974) and behavior during experiments (error rates). Experiments 1 and 2 show that it is neither misery nor happiness but “common unhappiness” (Freud, 1895, p. 322) that is desirable, because participants in a negative or positive mood did not perform well. This has been the case for decades in some therapeutic approaches which have recognized that being freed from misery better equips one to deal with life's adversities (Freud, 1895). People appear to find it easiest to process neutral (non-emotional) information (Experiments 1 and 2) but ideally sessions work with *hot cognitions* and elicit *key emotions* and *cognitions* (Safran and Greenberg, 1982; Beck, 1995). If neutral information becomes the focus of sessions, then sessions would elicit less key emotions and cognitions and turn into a nice chat which will be remembered pleasantly by the patient. Thereby the patient does not get overwhelmed with emotional material which will have a detrimental effect on reasoning. Instead the emotional material can be introduced bit by bit (e.g., as is the case in systematic desensitization in cognitive behavioral therapy).

It is worthwhile for patients to remember what has been discussed in sessions because new behaviors and alternative viewpoints which have been collaboratively developed in sessions may be easily forgotten, especially when the patient is suffering from a depression which often results in decreased concentration. Some therapists recommend that their patients take notes during sessions (Beck, 1995) but if only things that are easily remembered are discussed, this problem is circumvented. Therefore, if the patient wishes to get stabilized, non-emotional material may be best. If they want to work through distressing material however, it will not be possible to avoid emotional content. Hence emotions and cognitions are related and influence each other and one has to combine them according to what the goal is.

Thus far the key finding is that emotional state and content may interact to modulate logical reasoning. This is however only the case if (mood) state and (task) content are related (Experiment 3; spider-related content among spider phobics). But, this does so far not generalize to other contexts, since it could for example not be found in a sample with exam anxiety (Experiment 4; exam anxiety in combination with exam content). These ambiguities, the role of working memory and attentional processes need to be addressed in future studies in order to explain the influence of emotional content and emotion on human reasoning performance.

## Author contributions

Nadine Jung did the statistical analysis and wrote the paper. Christina Wranke designed and conducted the experiments, and did the statistical analysis. Kai Hamburger designed the experiments and wrote the paper. Markus Knauff designed the experiments and wrote the paper.

### Conflict of interest statement

The authors declare that the research was conducted in the absence of any commercial or financial relationships that could be construed as a potential conflict of interest.

## Footnotes

^1^For reasons of simplicity the term “emotion” is also used to represent “mood” (emotional state). The distinction between emotion and mood will only be pointed out were necessary.
